# Supplementary material for: NF-Y controls fidelity of transcription initiation at gene promoters through maintenance of the nucleosome-depleted region
Source: Nat Commun. 2019 Jul 11;10:3072. doi: 10.1038/s41467-019-10905-7 (PMC6624317; doi:10.1038/s41467-019-10905-7)
Supplement: Supplementary file 1 — Supplementary Information [file 41467_2019_10905_MOESM1_ESM.pdf]

SUPPLEMENTARY INFORMATION

NF-Y controls fidelity of transcription initiation at gene promoters through maintenance of the nucleosome-depleted region

Andrew J Oldfield, Telmo Henriques, Dhirendra Kumar, Adam B. Burkholder, Senthilkumar Cinghu, Damien Paulet, Brian Bennett, Pengyi Yang, Benjamin S. Scruggs, Christopher A. Lavender, Eric Rivals, Karen Adelman, Raja Jothi

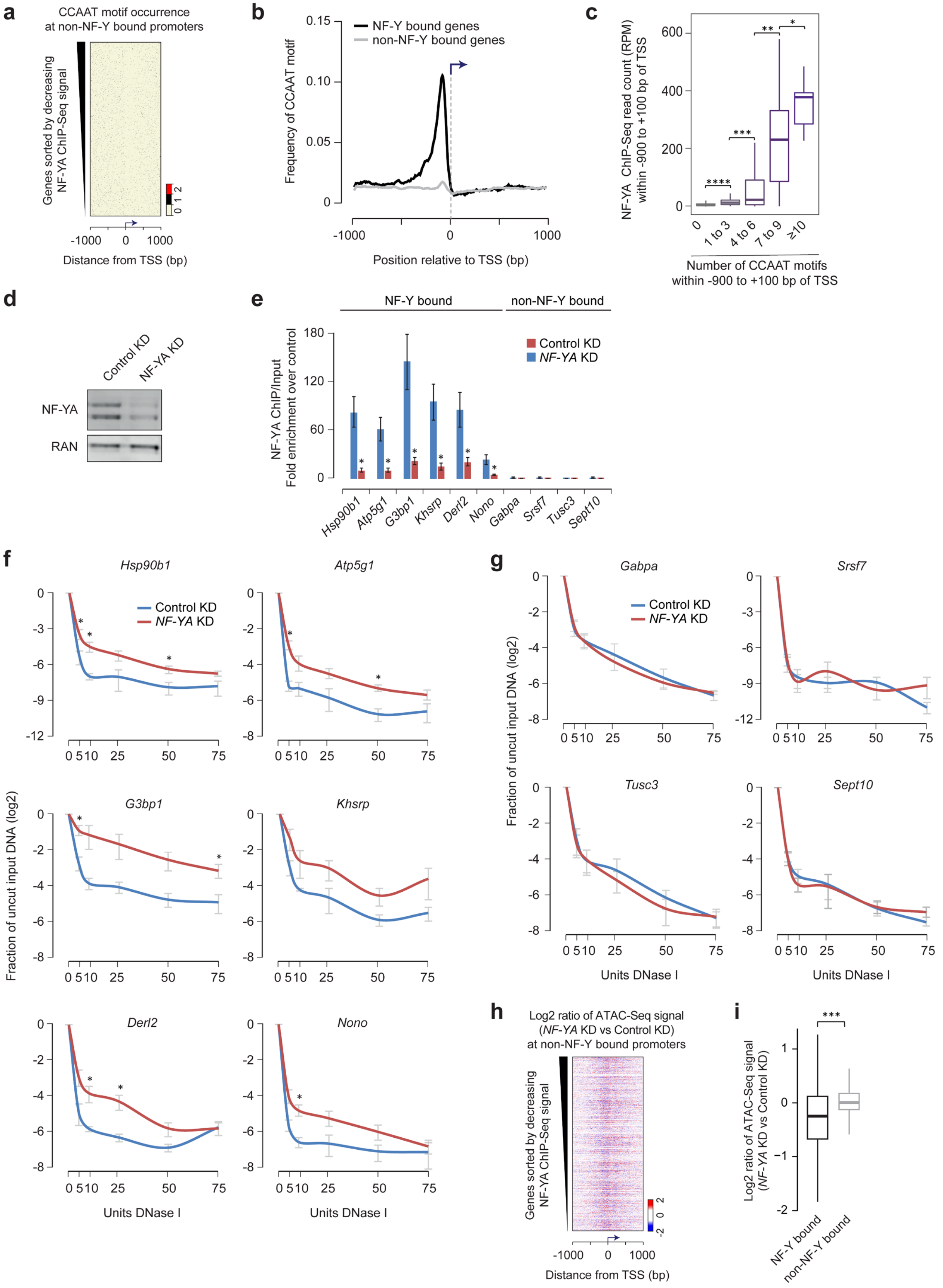
a
CCAAT motif occurrence at non-NF-Y bound promoters
Genes sorted by decreasing NF-YA ChIP-Seq signal
Distance from TSS (bp)
b
NF-Y bound genes
non-NF-Y bound genes
Frequency of CCAAT motif
Position relative to TSS (bp)
c
NF-YA ChIP-Seq read count (RPM) within -900 to +100 bp of TSS
Number of CCAAT motifs within -900 to +100 bp of TSS
0
1 to 3
4 to 6
7 to 9
≥10
d
Control KD
NF-YA KD
NF-YA
RAN
e
NF-Y bound
non-NF-Y bound
NF-YA ChIP/Input Fold enrichment over control
Control KD
NF-YA KD
Hsp90b1
Atp5g1
G3bp1
Khsrp
Derl2
Nono
Gabpa
Srsf7
Tusc3
Sept10
f
Fraction of uncut input DNA (log2)
Units DNase I
g
Gabpa
Srsf7
Tusc3
Sept10
h
Log2 ratio of ATAC-Seq signal (NF-YA KD vs Control KD) at non-NF-Y bound promoters
Genes sorted by decreasing NF-YA ChIP-Seq signal
Distance from TSS (bp)
i
Log2 ratio of ATAC-Seq signal (NF-YA KD vs Control KD)
NF-Y bound
non-NF-Y bound

Supplementary Figure 1 | NF-Y binding is required to maintain accessible chromatin at its target promoters

- **a,** CCAAT motif occurrence near TSSs of genes without promoter-proximal NF-Y binding.
- **b,** Frequency of CCAAT motif occurrence near TSSs of genes with (*n* = 3,056, black) or without *n* = 21,195, grey) promoter-proximal NF-Y binding in ESCs.
- **c,** Box plot showing the relationship between CCAAT motif occurrence within the promoter (-900 bp to +100 bp region relative to TSS, x-axis) and NF-YA ChIP-Seq read density (y-axis) for all annotated RefSeq genes. *P-value = 0.013, **P-value = 2.12E-22, ***P-value = 1.11E-88, ****P-value = 0 (Wilcoxon rank-sum test, two-sided)
- **d,** Western-blot analysis of NF-YA in control or *NF-YA* KD ESCs, 48 hr after siRNA transfection. Ran used as a loading control.
- **e,** ChIP-qPCR analysis of NF-Y binding sites in control or *NF-YA* KD ESCs. Error bars, SEM of three to five Biological replicates.
- **f,g** DNase I hypersensitivity and qPCR analysis of promoters with (f) or without (g) NF-Y binding in control (blue) and *NF-YA* KD (red) ESCs. Error bars, SEM of three biological replicates. *P-value < 0.05 (Student's t-test, two-sided).
- **h,** Relative change (log2) in chromatin accessibility, as measured using ATAC-Seq, near TSSs of genes without promoter-proximal NF-Y binding in *NF-YA* KD vs control KD ESCs.
- **i,** Box plot showing the distribution of foldchanges in ATAC-Seq signal (in NF-YA KD vs control KD ESCs) within the upstream proximal-promoter regions (-150 bp to -50 bp). ***P-value = 2.86E-209 (Wilcoxon rank-sum test, two-sided)

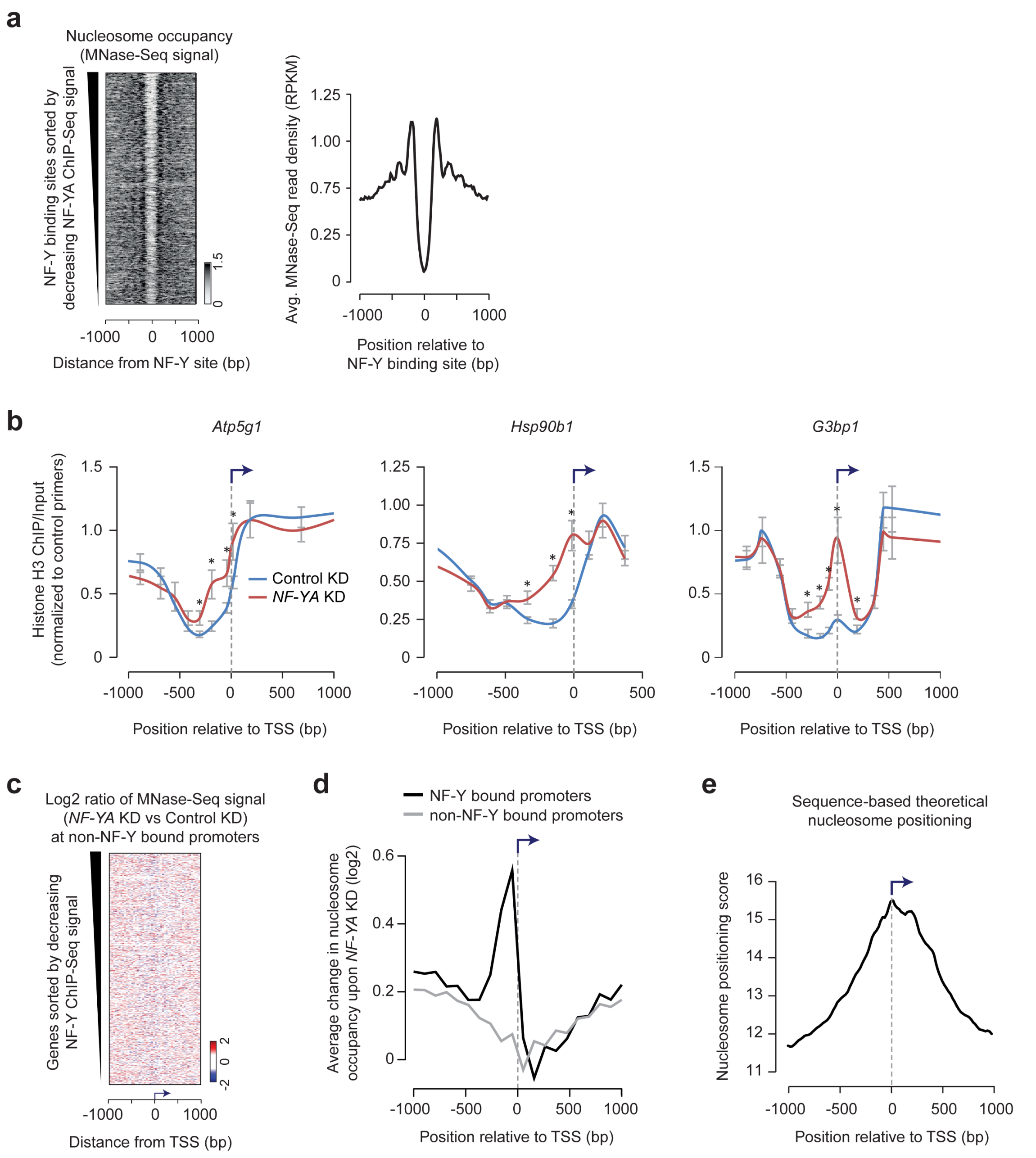

Supplementary Figure 2 | NF-Y binding protects nucleosome-depleted region from nucleosome encroachment

- **a,** *Left*: Nucleosome occupancy, as measured using MNase-Seq, at all NF-Y binding sites (*n* = 5,359) in ESCs. *Right*: Average nucleosome occupancy (y-axis) at NF-Y binding sites. RPKM, reads per million mapped reads.
- **b,** ChIP-qPCR analysis of Histone H3 occupancy at candidate gene promoters with NF-Y binding in control or *NF-YA* KD ESCs. Error bars, SEM of three replicates.

c, Relative change in nucleosome occupancy (gain, red; loss, blue) near TSSs of genes without promoter-proximal NF-Y binding in *NF-YA* KD vs control KD ESCs.

d, Average change in nucleosome occupancy (y-axis) in *NF-YA* KD vs control KD ESCs near TSSs of genes with (black) or without (gray) promoter-proximal NF-Y binding.

e, Density plot showing average theoretical nucleosome positioning, predicted based on DNA sequence composition, near TSSs of genes with promoter-proximal NF-Y binding in ESCs.

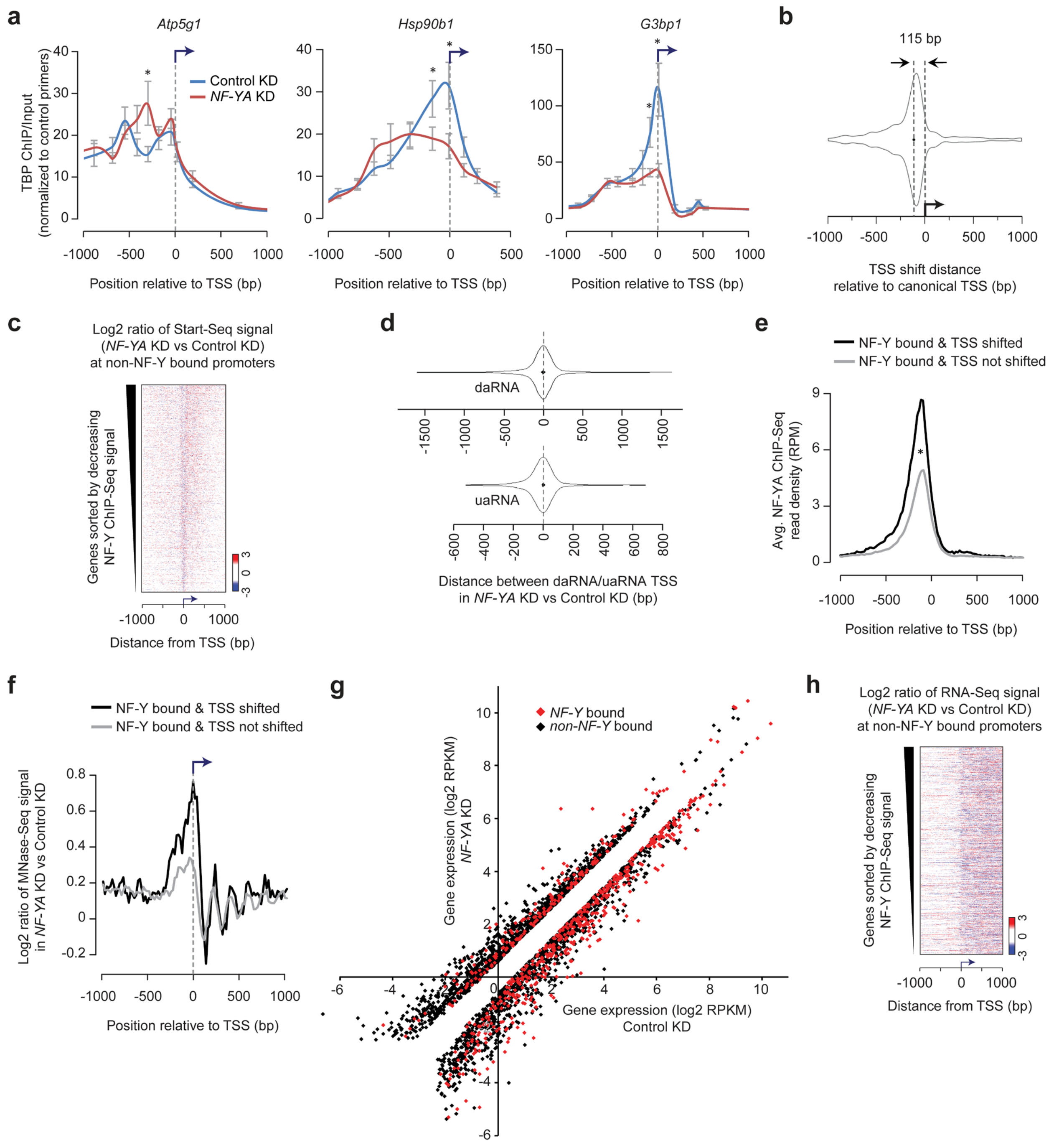

Supplementary Figure 3 | NF-Y binding influences PIC positioning and TSS selection

- **a,** ChIP-qPCR analysis of TBP occupancy at candidate gene promoters with promoter-proximal NF-Y binding in control (blue) or *NF-YA* KD (red) ESCs. Error bars, SEM of three replicates.
- **b,** Violin plot showing the distribution of TSS shift distance, as measured using Start-Seq, in *NF-YA* KD vs control KD ESCs. Median shift distance = 115bp.

c, Relative fold-change (log2) in Start-Seq signal (red, gain; blue, loss) in *NF-YA* KD vs control KD cells near TSSs of genes without promoter-proximal NF-Y binding.

d, Violin plot showing the distribution of distance by which the TSS of upstream (or downstream) anti-sense RNA (uaRNA or daRNA, respectively) shifted its position, as measured using Start-Seq, in *NF-YA* KD vs control KD ESCs. Median indicated by dotted grey line.

e, Average NF-YA occupancy (y-axis), as measured using ChIP-Seq, near TSSs of genes with promoter-proximal NF-Y binding that exhibit (black, *n* = 538) or do not exhibit (grey, *n* = 2,518) an ectopic TSS (TSS shift) in *NF-YA* KD vs control KD ESCs. RPM, reads per million mapped reads.

f, Relative change in nucleosome occupancy (gain, red; loss, blue) near TSSs of genes with promoter-proximal NF-Y binding that exhibit (black, *n* = 538) or do not exhibit (grey, *n* = 2,518) an ectopic TSS (TSS shift) in *NF-YA* KD vs control KD ESCs.

g, Scatter plot showing the (RNA-Seq) expression of NF-Y bound (red, *n* = 660) and non-NF-Y bound (black, *n* = 2,389) genes in control (x-axis) and NF-YA KD (y-axis) ESCs. Only genes whose expression is significantly different (>1.5-fold) in NF-YA KD vs control cells are shown.

h, Relative fold-change (log2) in RNA-Seq signal (red, gain; blue, loss) in *NF-YA* KD vs control KD cells near TSSs of genes without promoter-proximal NF-Y binding.

a

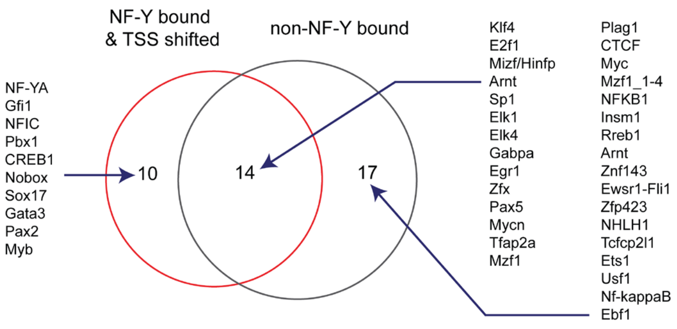

b

Rank	JASPAR Matrix_ID	TF Name	Motif	Occurrence	Chi-squared	P-value	P-value adjusted for multiple testing
1	MA0060.1	NF-YA	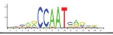	471/538	3059.89	1.12E-183	1.32E-181
2	MA0038.1	Gfi1	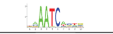	326/538	1928.45	7.36E-50	4.34E-48
3	MA0161.1	NFIC	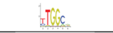	439/538	1676.22	3.86E-28	1.35E-26
4	MA0070.1	Pbx1	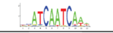	136/538	1515.05	7.27E-17	1.23E-15
5	MA0018.2	CREB1	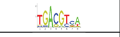	175/538	1307.45	3.14E-06	2.65E-05
6	MA0125.1	Nobox	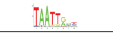	101/538	1289.13	1.54E-05	1.21E-04
7	MA0078.1	Sox17	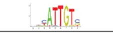	226/538	1282.05	2.76E-05	2.04E-04
8	MA0037.1	Gata3	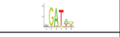	286/538	1271.15	6.61E-05	4.33E-04
9	MA0067.1	Pax2	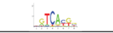	182/538	1237.61	7.66E-04	4.52E-03
10	MA0100.1	Myb	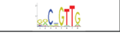	141/538	1209.32	4.55E-03	2.24E-02

c

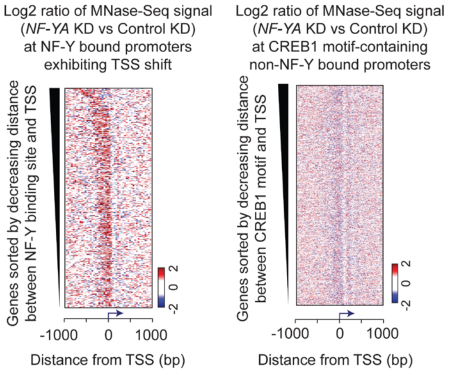

d

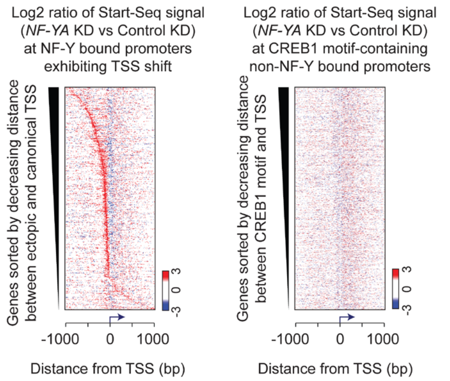

Supplementary Figure 4 | Analysis of TF binding motifs enriched within the promoters of NF-Y bound genes exhibiting a TSS shift.

- **a,** Venn diagram summarizing the list of TF binding motifs (source: JASPAR database) enriched within the promoter sequences (200 bp upstream of TSS) of NF-Y bound genes exhibiting a TSS shift and non-NF-Y bound genes.
- **b,** Summary statistics of TF binding motifs enriched within the promoter sequences (200 bp upstream of TSS) of NF-Y bound genes exhibiting a TSS shift.
- **c,** Comparison of changes in nucleosome occupancy (NF-YA KD vs control KD) near TSSs of NF-Y bound genes exhibiting a TSS shift (*left*) vs non-NF-Y bound genes containing a CREB1 binding motif within 200 bp upstream of TSS (*right*).
- **d,** Comparison of changes in Start-Seq signal (NF-YA KD vs control KD) near TSSs of NF-Y bound genes exhibiting a TSS shift (*left*) vs non-NF-Y bound genes containing a CREB1 binding motif within 200 bp upstream of TSS (*right*).

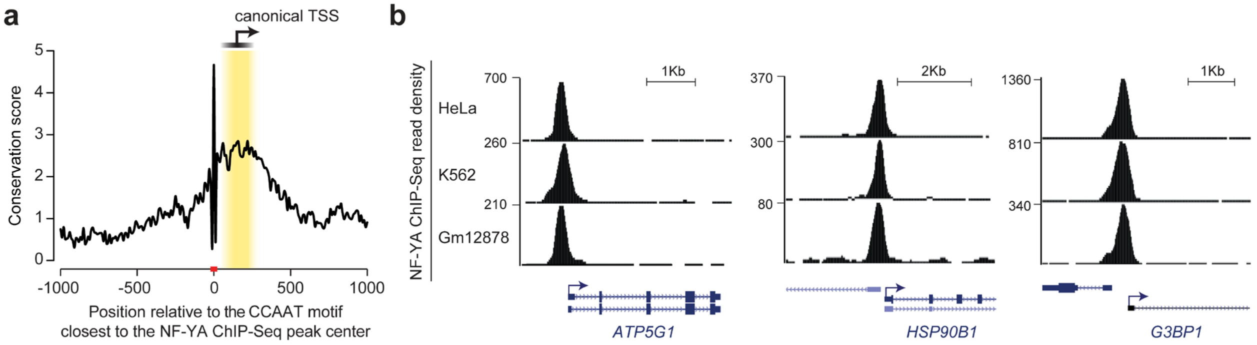

Supplementary Figure 5 | Conservation of NF-Y binding in mammals

- **a,** Average sequence conservation in mammals, as computed using PhyloP, of the 2 Kb region centered on the CCAAT motif closest to the NF-Y peak center. Only genes with promoter-proximal NF-Y binding that exhibit an ectopic TSS were used (n = 538).
- **b,** Genome browser shots of genes, which bind NF-Y within promoter-proximal regions in mouse ESCs (see Figure 2), showing promoter-proximal NF-Y binding in human normal (Gm12878) and cancer cells (HeLa and K562). Human NF-YA ChIP-Seq data from ENCODE (GSE31477) was used.

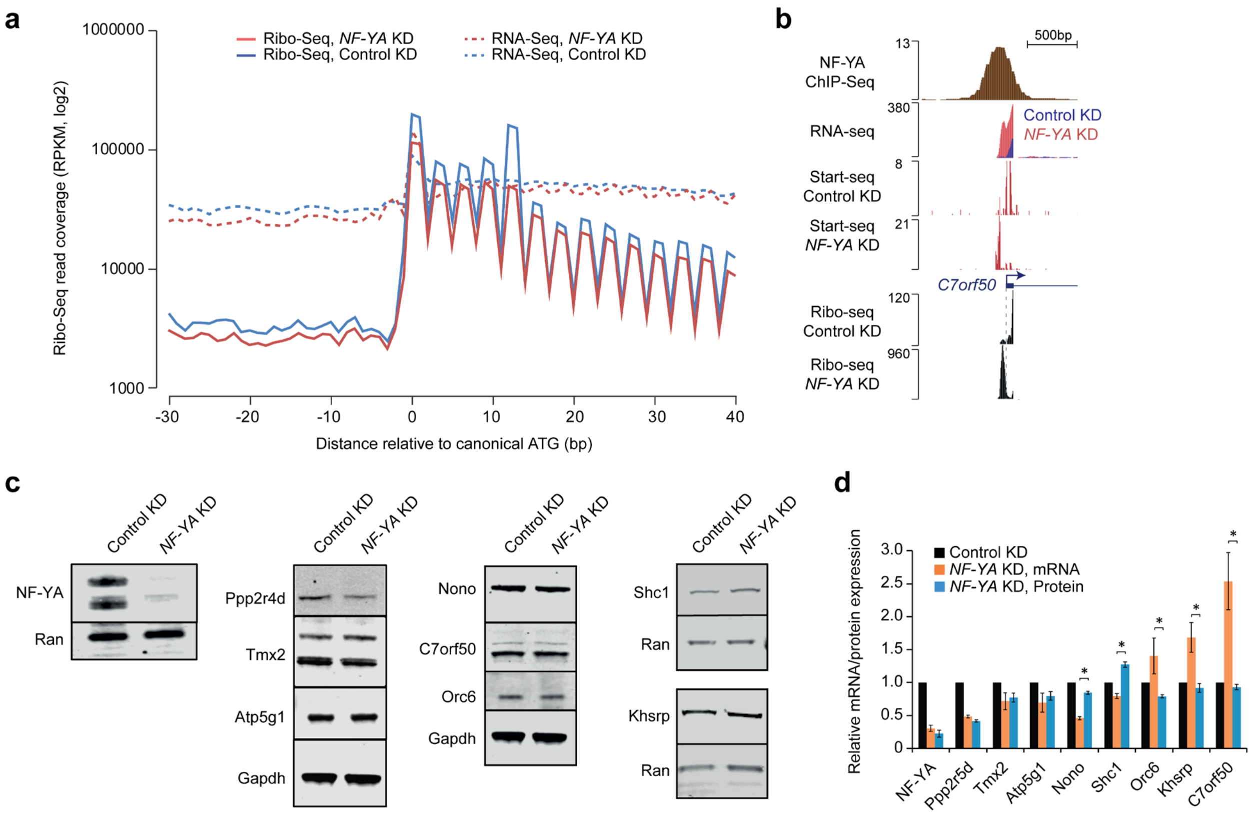

Supplementary Figure 6 | Transcripts originating from ectopic TSSs in NF-Y depleted cells undergo translation

- **a,** Ribo-seq and RNA-Seq read coverage, centered on the canonical start codon (ATG), for all genes in control and *NF-YA* KD ESCs. Triplet phasing, beginning at the canonical start codon, is observed for Ribo-Seq but not RNA-Seq data. RPKM, reads per kilobase per million mapped reads.
- **b,** Genome browser shot of NF-Y target gene *C7orf50* (3110082I17Rik) showing ribosome-protected RNA expression, as measured using Ribo-Seq, in control (blue) and *NF-YA* KD (red) ESCs. Also shown are tracks for RNA-Seq and Start-Seq in control and *NF-YA* KD ESCs.
- **c,** Western-blot analysis of NF-YA, Ppp2r5d, Tmx2, Atp5g1, Nono, C7orf50, Orc6, Shc1 and Khsrp in control and NF-YA knock-down (KD) ESCs 48h after siRNA transfection. Ran or Gapdh used as loading controls. Representative images are shown.
- **d,** Relative changes in gene expression (mRNA, orange) and protein levels (blue) in *NF-YA* KD vs control ESCs. Protein levels were determined using Western-blot analysis (see Figure S4C), quantified by Licor Image Studio® software. mRNA data normalized to *Actin*, *HAZ* and *TBP*. Protein data normalized to Ran or Gapdh. Error bars, SEM of three to five biological replicates. *P-value < 0.00002 (Student's t-test, two-sided)
