# Supplementary material for: NF-Y controls fidelity of transcription initiation at gene promoters through maintenance of the nucleosome-depleted region
Source: Nat Commun. 2019 Jul 11;10:3072. doi: 10.1038/s41467-019-10905-7 (PMC6624317; doi:10.1038/s41467-019-10905-7)
Supplement: Supplementary file 2 — Description of Additional Supplementary Files [file 41467_2019_10905_MOESM2_ESM.pdf]

Description of Additional Supplementary Files

File name: Supplementary Data 1

Description: List of NF-Y bound genes with a TSS shift upon NF-YA KD that contain translation start codon AUG within the ectopically transcribed region.

File name: Supplementary Data 2

Description: List of primers used for RT-qPCR, DNase-qPCR, ChIP-qPCR, and MNase-qPCR studies
